# Integrative Mendelian Randomization and Proteomic Analyses Reveal Genetically Supported Candidate Causal Biomarkers for Non-Traumatic Osteonecrosis of the Femoral Head

**DOI:** 10.21627/zrn0dg78

**Published:** 2026-07-06

**Authors:** Zhangzheng Wang, Liang Mo, Wei He, Youwen Liu, Csaba Matta, Yuhao Liu

**Affiliations:** The First Affiliated Hospital of Guangzhou University of Chinese Medicine, Guangzhou, China; Department of Anatomy, Histology and Embryology, Faculty of Medicine, University of Debrecen, Debrecen, Hungary; The First Affiliated Hospital of Guangzhou University of Chinese Medicine, Guangzhou, China; The First Affiliated Hospital of Guangzhou University of Chinese Medicine, Guangzhou, China; Luoyang Orthopedic-Traumatological Hospital of Henan Province, Luoyang, China; Department of Anatomy, Histology and Embryology, Faculty of Medicine, University of Debrecen, Debrecen, Hungary; Department of Biomedical Materials Science, Graduate School of JABA, Wonkwang University, Iksan, Republic of Korea; Integrated Omics Institute, Wonkwang University, Iksan, Republic of Korea; The First Affiliated Hospital of Guangzhou University of Chinese Medicine, Guangzhou, China; Luoyang Orthopedic-Traumatological Hospital of Henan Province, Luoyang, China; Guangdong Clinical Research Academy of Chinese Medicine, Guangzhou, China

**Keywords:** Mendelian randomization, proteomics, non-traumatic osteonecrosis of the femoral head, causal biomarkers, eQTL

## Abstract

**INTRODUCTION:**

Non-traumatic osteonecrosis of the femoral head (NONFH) is a highly disabling orthopedic disorder characterized by an insidious onset and significant clinical challenges. Although quantitative proteomic profiling of necrotic and adjacent healthy bone tissues has elucidated aspects of disease mechanisms, fundamental limitations remain in comprehensively understanding NONFH pathogenesis.

**OBJECTIVES:**

This study aimed to identify causal biomarkers for NONFH by integrating differentially expressed proteins (DEPs) with transcriptome-wide association study (TWAS) and Mendelian randomization (MR) analyses.

**METHODS:**

Data-independent acquisition (DIA)-based proteomics was performed on necrotic, sclerotic, and healthy bone tissues to identify DEPs. TWAS-prioritized genes were subjected to MR using blood and musculoskeletal tissue-specific expression quantitative trait loci (eQTLs). Reliable causal relationships were retained by excluding pleiotropy or heterogeneity and requiring consistent directions across methods (IVW, WME, and MR-Egger) and multiple analyses. The functional relevance and cell-type specificity of these causal biomarkers were examined using published single-cell RNA sequencing data.

**RESULTS:**

Proteomic analysis revealed a total of 846 DEPs. MR identified five causal genes in blood (*ACOT1*, *ALDH1A1*, *DAG1*, *STEAP4*, *LIPA*) and four causal genes in musculoskeletal tissue (*ACOT1*, *PPIC*, *TSC22D4*, *LIPA*). *ACOT1* and *ALDH1A1* exhibited protective effects (*β* < 0), whereas *DAG1*, *STEAP4*, *LIPA*, *PPIC*, and *TSC22D4* were associated with increased disease risk (*β* > 0).

**CONCLUSION:**

Seven genes (*ACOT1*, *LIPA*, *PPIC*, *TSC22D4*, *ALDH1A1*, *DAG1*, and *STEAP4*) demonstrating both proteomic dysregulation and genetic causality may serve as novel biomarkers or therapeutic targets for NONFH. These findings provide a foundation for further mechanistic studies and hold potential for advancing diagnosis and treatment strategies for NONFH.

## Non-Specialist Summary

We studied why people develop a severe hip disease called non‑traumatic osteonecrosis of the femoral head, in which bone tissue gradually dies. By comparing damaged and healthy bone and linking these findings with human genetic information, we identified several molecules that may directly influence the development of this condition. Our results may help improve early detection and guide future treatments for people at risk of this disease.

## 1. Introduction

Osteonecrosis of the femoral head (ONFH) is a debilitating orthopedic condition characterized by progressive bone destruction and high disability rates. Although epidemiological studies have identified steroid use and alcohol abuse as major risk factors [1], the underlying molecular mechanisms of non-traumatic osteonecrosis of the femoral head (NONFH) remain elusive, posing significant challenges for its effective diagnosis and treatment.

Recent advances in mass spectrometry (MS)-based proteomics have enabled high-throughput detection and quantification of proteins in biological samples, offering valuable insights into disease pathogenesis [2]. This approach facilitates targeted investigation of proteomic differences between localized pathological lesions and adjacent healthy tissues, such as necrotic versus healthy bone. Despite the identification of differentially expressed proteins (DEPs), several limitations persist. Proteomic variations may result from methodological and technical inconsistencies [3], confounding clinical factors such as underlying comorbidities, medication effects, or secondary consequences of treatment, such as glucocorticoid therapy. Additionally, certain protein expression changes may represent downstream, non-causal responses to the pathological microenvironment rather than genuine drivers of disease. Conventional bioinformatics analyses, such as pathway enrichment and protein–protein interaction networks, although useful for interpreting proteomic data, often primarily emphasize established biological pathways, potentially overlooking novel pathogenic mediators implicated in NONFH.

To address these limitations, the integration of transcriptome-wide association studies (TWASs) and Mendelian randomization (MR) provides a robust framework for identifying causal biomarkers. Both methods interrogate the relationship between genetic variation and disease phenotypes using summary statistics of genome-wide association studies (GWASs). TWASs utilize expression quantitative trait loci (eQTLs) to prioritize genes associated with disease phenotypes at the transcriptional level [4]. MR, a widely adopted statistical framework, employs genetic variants as instrumental variables (IVs), infers causal relationships between exposures (e.g., transcript levels) and outcomes (e.g., ONFH), thereby mitigating confounding factors and minimizing reverse causation [5]. Combining these complementary approaches enables the rigorous prioritization of DEPs by linking proteomic results directly to genetic causality and, consequently, enables the nomination of candidate proteins with putative causal roles in osteonecrosis.

Given the tissue-specific nature of protein expression, incorporating bone-derived eQTL data further refines the precision of causal inference, which is particularly critical for NONFH, where emerging therapeutic strategies such as intraosseous drug delivery target local bone tissue compartments. In this study, we introduce an integrative analytical pipeline that combines MS-based proteomics and TWAS and MR analyses to identify key proteins exhibiting both proteomic dysregulation and genetic evidence for causality. Our findings are expected to facilitate the discovery of novel biomarkers and therapeutic targets, with promising translational implications for improved diagnosis and treatment of NONFH.

## 2. Materials and Methods

### 2.1. Study Population

This study was conducted at the First Affiliated Hospital of Guangzhou University of Chinese Medicine with approval from the institutional Ethics Committee (approval number: K-2023-181). All procedures adhered to the principles of the Declaration of Helsinki and relevant regulatory guidelines. Written informed consent was obtained from all participants prior to enrollment.

The cohort comprised patients diagnosed with NONFH who subsequently underwent total hip arthroplasty (THA). Comprehensive preoperative imaging data were collected for all participants and independently evaluated by two experienced radiologists to determine the extent and stage of osteonecrosis.

### 2.2. Inclusion and Exclusion Criteria

Participants were eligible if they had a confirmed diagnosis of NONFH according to the Guidelines for Clinical Diagnosis and Treatment of Osteonecrosis of the Femoral Head in Adults (2019 version) [6] and were aged between 18 and 55 years. Patients were excluded if they had a history of traumatic hip injury or other metabolic bone disorders, previous hip-preserving surgical interventions such as bone grafting or core decompression, use of glucocorticoids or anti-osteoporotic agents within 3 months prior to THA, or severe comorbidities affecting cardiovascular, cerebrovascular, renal, or hematological systems.

### 2.3. Proteomic Analysis

Protein extraction from femoral head bone tissues was performed according to established protocols [7]. (1) Femoral head specimens were collected during THA. Necrotic, sclerotic, and healthy bone tissues were dissected (Figure 1). (2) The samples were ground in liquid nitrogen, and the powder was transferred into a 5 mL Eppendorf tube. (3) LSDT protein lysis buffer [4% sodium dodecyl sulfate (SDS), 100 mM Tris-HCl, 100 mM dithiothreitol (DTT), pH 8.0] was then added. The mixture was boiled for 5 min, homogenized by ultrasonication in an ice bath for 5 min, and centrifuged at 14,000*g* for 30 min. The supernatant was filtered and stored at –80°C. (4) For each sample, 100 µg of protein was collected for enzymatic hydrolysis. Peptides were desalted using a Strata X column and vacuum-dried prior to mass spectrometric analysis.

**Figure 1.**
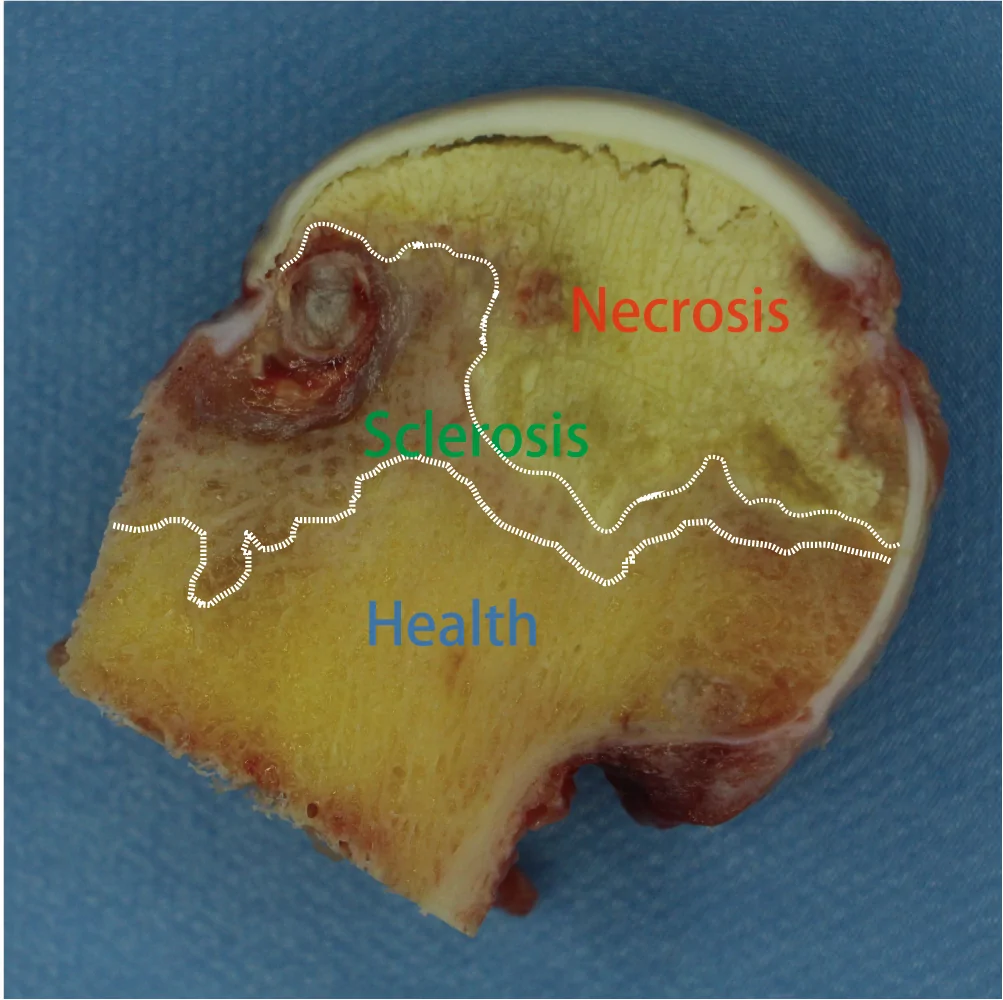
Macroscopic features of osteonecrosis of the femoral head (ONFH). Cross-sectional macroscopic view of a femoral head specimen obtained during total hip arthroplasty, illustrating the distinct anatomical regions sampled for data-independent acquisition (DIA)-based proteomic analysis: yellowish-white necrotic bone, gray sclerotic bone, and yellow healthy bone.

Peptide prefractionation was performed using high-pH reversed-phase (RP) chromatography on a Shimadzu LC-20AB HPLC system equipped with a Gemini C18 column (5 µm, 4.6 × 250 mm) (Phenomenex, Torrance, CA, USA). The peptide mixture was diluted with mobile phase A [5% acetonitrile (ACN), pH 9.8] and separated with the following gradient at a flow rate of 1 mL/min: 5% phase B [95% ACN, pH 9.8] for 10 min, 5–35% phase B over 40 min, 35–95% phase B in 1 min, held at 95% phase B for 3 min, and re-equilibrated at 5% phase B for 10 min. Elution was monitored at 214 nm, and fractions were collected every minute. A total of 10 fractions were pooled and freeze-dried.

The dried peptide samples were reconstituted in mobile phase A (0.1% formic acid [FA] in H_2_O), centrifuged at 20,000*g* for 10 min, and the supernatant was collected for injection. Separation was carried out using a Bruker NanoElute. Peptides were first trapped and desalted on a precolumn, then separated on a self-packed C18 analytical column (75 μm i.d. × 25 cm, 1.8 μm particles) packed with Xtimate UHPLC C18 material (Welch Materials, Guangzhou, China). The flow rate was 300 nL/min with the following gradient: 2% phase B (0.1% FA in ACN) from 0 min, 2–22% phase B over 45 min (0–45 min), 25–35% phase B in 5 min (45–50 min), 35–80% phase B in 5 min (50–55 min), and held at 80% phase B for 5 min (55–60 min). The eluted peptides were directly introduced into the mass spectrometer for analysis.

In this study, we used an integrated approach combining data-dependent acquisition (DDA) and data-independent acquisition (DIA) [8]. For DDA analysis, peptides separated by liquid chromatography were ionized using a nanoESI source and analyzed on a Bruker timsTOF Pro mass spectrometer operated in DDA mode. The instrument parameters were set as follows: ion source voltage, 1.6 kV; MS1 scanning range, 100–1700 m/z; ion mobility range, 0.6–1.60 V·s/cm^2^; MS2 scanning range, 100–1700 m/z. Precursor ions selected for MS2 fragmentation had charge states from 0 to 5+, with the 10 most-intense ions exceeding an intensity threshold of 10,000 and a minimum intensity of 2,500 included for fragmentation. The cumulative scan time was set to 100 ms for both MS1 and MS2. CID was used as the fragmentation mode, and fragment ions were detected in the Time-of-Flight mass (TOF) analyzer. A dynamic exclusion time of 30 s was applied. DDA data were processed using the Andromeda search engine integrated in MaxQuant, with protein identification performed against the UniProtKB/Swiss-Prot *Homo sapiens* database. The resulting spectral library was subsequently used for DIA analysis.

For DIA, the timsTOF Pro mass spectrometer was operated in data-independent acquisition with parallel accumulation serial fragmentation (diaPASEF) mode. The key parameters were configured as follows: ion source voltage, 1.6 kV; ion mobility range, 0.6–1.60 V·s/cm^2^; MS1 scanning range, 302–1077 m/z, with a minimum intensity threshold of 2500. The m/z range was divided into four steps, with each step further segmented into eight windows, resulting in a total of 32 windows for continuous fragmentation and data acquisition. CID was used as the fragmentation mode with the collision energy set to 10 eV, and each window had a mass width of 25. The cycle time of a DIA scan was 3.3 s.

The DIA data were processed using Spectronaut [9] with the established spectral library from DDA. The key settings were as follows: For retention-time alignment, the dynamic indexed retention time (iRT) method was applied using the default iRT peptide set provided by Spectronaut. To control the false discovery rate, a decoy database was generated by the scrambled method. The mProphet algorithm [10] was then employed to discriminate correct from incorrect peptide-spectrum matches on the basis of a set of quality features, and peptide as well as protein-level identifications were filtered at a 1% *Q*-value cutoff. Local normalization was applied to correct for systematic run-to-run variations. Finally, for protein quantification and differential analysis, protein summarization was performed using the *MSstats* package (version 4.16.1) [11] in R (version 4.5.1).

After proteins with missing values in ≥30% of samples were removed, the remaining missing values were imputed using the median value of the respective protein across all samples within the same region (Necrosis, Sclerosis, and Health). The data were normalized using the *justvsn* function from the *vsn* package (version 3.76.0). This study included nine hips, with three distinct regions measured per hip, thus constituting a repeated measures design. To account for the non-independence of regional measurements originating from the same hip, a mixed-model analysis approach provided by the *Limma* package (version 3.64.3) was adopted. The specific workflow was as follows: First, the *duplicateCorrelation* function was used to estimate the within-block consensus correlation, using the hip ID as the blocking variable. This estimated correlation value was then incorporated into the linear model fitting process to accurately adjust for the correlation introduced by the repeated measurements within hips when assessing expression differences between regions. Differential analysis was performed with thresholds set at an adjusted *p*-value (adj.*p*) < 0.05 (Benjamini–Hochberg correction) and an absolute log2 fold change (FC) ≥ 0.58. Proteins meeting these criteria were identified as differentially expressed proteins (DEPs).

### 2.4. Mendelian Randomization Analyses

TWAS is a statistical approach that integrates reference data on genetic variants (single nucleotide polymorphisms, SNPs) and gene expression with summary statistics from GWASs to identify associations between genetically mediated gene expression and complex traits [4]. In our study, we first identified a set of DEPs relevant to osteonecrosis through prior analyses. Therefore, we used integrated TWAS results from the Transcriptome-Wide Association Studies Atlas (ngdc.cncb.ac.cn/twas/) to interrogate this specific set of DEP-encoding genes, screening for those that also exhibit TWAS significance. This two-tiered approach enabled us to prioritize genes that demonstrate both proteomic alterations and transcriptional regulation relevant to osteonecrosis. These prioritized genes were then employed as exposures in MR analyses to investigate their potential causal roles.

MR is a method that uses genetic variants in the form of SNPs from GWAS summary data to infer causal effects of an exposure on an outcome. IVs for MR analysis were selected by integrating eQTLs associated with the exposure genes and GWAS summary statistics for osteonecrosis. Both TWAS and MR reduce bias from postnatal environmental factors by leveraging genetic variation, which is particularly valuable for osteonecrosis, where environmental confounders such as medication use can obscure true causal relationships in observational studies. However, MR provides more stringent causal estimates by explicitly testing exposure–outcome relationships, thereby enhancing the reliability and clinical translational potential of biomarkers identified through this approach. Thus, MR served as the final causal validation step, testing whether the TWAS-prioritized genes directly influence osteonecrosis.

eQTLs data for these exposure genes were sourced from three datasets: the eQTLGen consortium (whole blood cis-eQTL from 31,684 individuals [12]), the Genotype-Tissue Expression (GTEx) project for musculoskeletal and whole blood tissues (retrieved using the *xQTLbiolinks* R package (version 1.7.3) [13]). Stringent cis-eQTL selection criteria included genomic location within ±1 Mb of gene boundaries [14], statistical significance at *p* < 5 × 10^–8^, and adequate instrument strength with *F*-statistic ≥ 10.

Because alcohol or steroids have systemic effects, key genetic alterations induced by these factors that contribute to the development of NONFH may also be observable in whole blood. Therefore, we incorporated whole blood data as a reference.

GWAS summary statistics for osteonecrosis outcomes were obtained from UK Biobank (*n* = 361,141) [15] and FinnGen Consortium (1788 cases and 473,264 controls) [16]. SNPs significantly associated with outcomes at *p* < 5 × 10^–8^ were excluded from the analysis to reduce confounding. MR analyses employed the *TwoSampleMR* R package (version 0.7.0) with linkage disequilibrium (LD) clumping at *r*^2^ < 0.3 within 100-kb windows [17], and minor allele frequency (MAF) > 0.01. The inverse-variance weighted (IVW) method served as the primary analytical approach [18], with sensitivity analyses assessing heterogeneity via Cochran’s *Q*-test and horizontal pleiotropy by Mendelian randomization–Egger regression (MR–Egger) intercept test. IVW results demonstrating significant (*p* < 0.05) heterogeneity or pleiotropy were excluded.

The IVW method is the core approach for causal effect estimation in two-sample MR. Hence, after excluding results with significant heterogeneity or pleiotropy, we retained only those genes for which the IVW results were significant. To further ensure robustness, we also applied the weighted median estimator (WME) and MR–Egger for additional validation. Only genes for which the *β* values from IVW, WME, and MR–Egger were consistently positive or negative across every analysis were retained. Additionally, because the GWAS data for osteonecrosis contain a relatively low proportion of cases, we performed multiple analyses using data from different sources to reduce potential bias. On this basis, we further filtered the genes, retaining only those for which the IVW method showed consistent effect directions (*β* values) across multiple analyses. These genes exhibit consistency in the direction of effect across both multiple methods and repeated comparisons and are ultimately designated as candidate causal biomarkers.

The significance threshold was adjusted using Bonferroni correction based on the number of genes with valid instrumental variables in each analysis (Figure 2).

**Figure 2.**
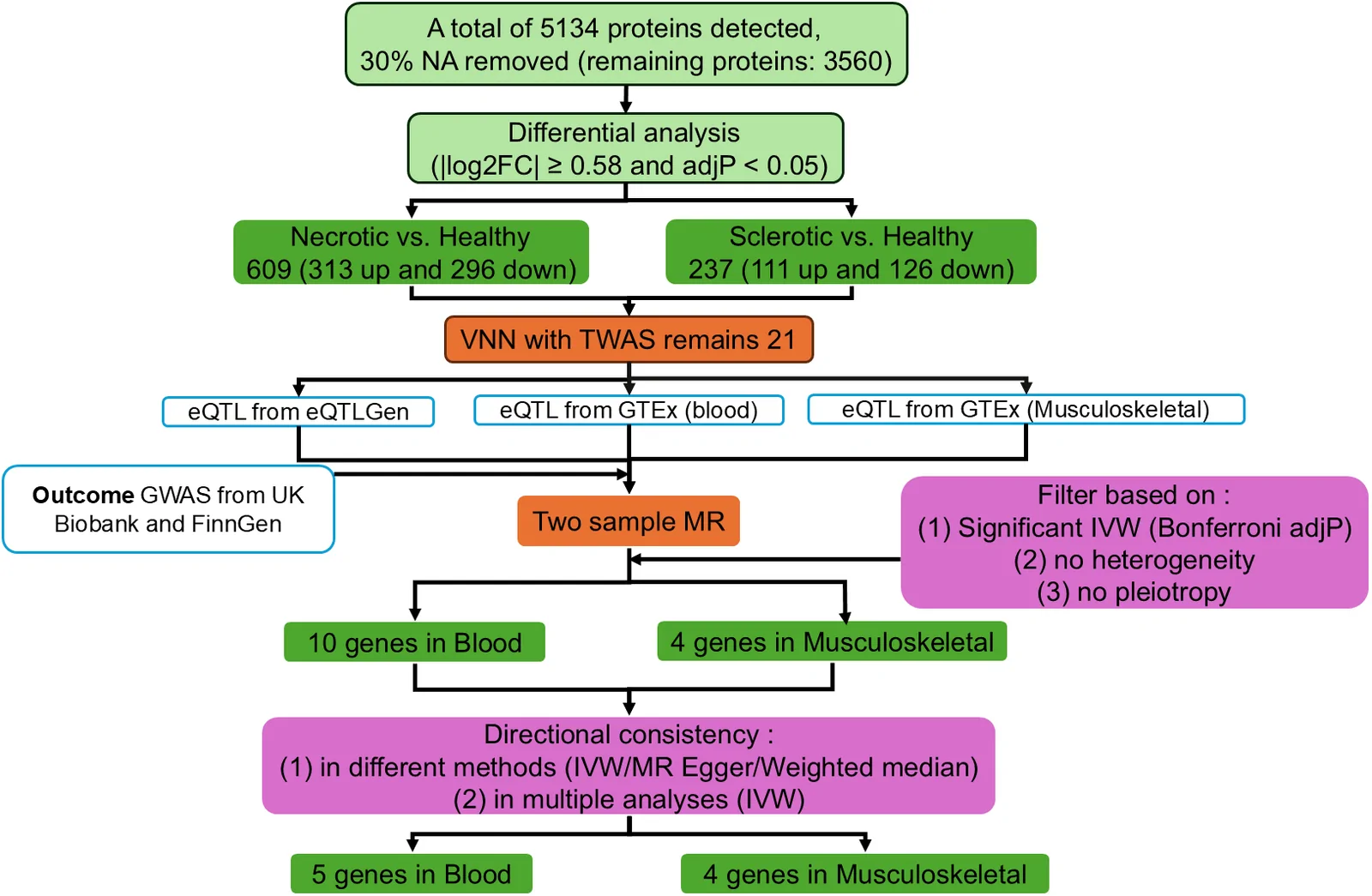
Analytical framework flowchart.

### 2.5. Single-Cell RNA Sequencing Analyses

To further explore the functional roles of the identified causal biomarkers in NONFH pathogenesis and progression, analyses were performed using a publicly available single-cell RNA sequencing (scRNA-seq) dataset derived from femoral head bone tissue of patients undergoing THA [19]. To maximize cell yield while managing computational constraints, two samples per group were selected: ARCO stage III (ONFH2, ONFH3), ARCO stage IV (ONFH5, ONFH6), and osteoarthritis controls (OA2 and OA3).

Data processing followed the workflow described in the original study [19], implemented using the *Seurat* R package (version 5.4.0). Quality control excluded cells with fewer than 500 unique molecular identifiers (UMIs), fewer than 250 detected genes, mitochondrial gene expression exceeding 25% of total expression, or a gene-to-UMI ratio less than 0.8. The data from the selected samples were merged, batch-corrected, and normalized using the *SCTransform* and *IntegrateData* functions. Unsupervised clustering used 40 principal components with a clustering resolution of 0.5; clusters were manually annotated.

Cluster-specific marker genes were identified with the *FindAllMarkers* function, retaining positive markers with log2FC ≥ 0.25. Cell–cell communication analysis was conducted using the *CellChat* R package (version 1.6.1) to infer intercellular signaling interactions.

## 3. Results

### 3.1. Demographic Characteristics

After the inclusion and exclusion criteria were applied, seven patients (nine hips) with NONFH were enrolled in the study. The cohort comprised three males and four females, with a mean age of 36.29 ± 5.87 years. Among the nine affected hips, three were classified as ARCO stage II and six as ARCO stage III (Table 1).

**Table 1.** Basic characteristics of the study subjects.

Patient ID	Gender	Age (years)	Side	ARCO Stage
1	male	43	right	III
2	male	30	right & left	II &II
3	female	41	left	III
4	female	35	right & left	II & III
5	female	27	left	III
6	male	35	left	III
7	female	43	right	III

### 3.2. DIA-Based Proteomic Profiling

DIA proteomics identified a total of 5134 proteins across all samples. To ensure analytical robustness, proteins with missing values in more than 30% of samples were excluded, yielding a final dataset of 3560 proteins for downstream analyses. Variance-stabilizing normalization (VSN) was applied to reduce technical variability (Figure 3A), and principal component analysis (PCA) demonstrated clear separation between necrotic, sclerotic, and healthy bone tissues, with minimal intra-group variation (Figure 3B).

**Figure 3.**
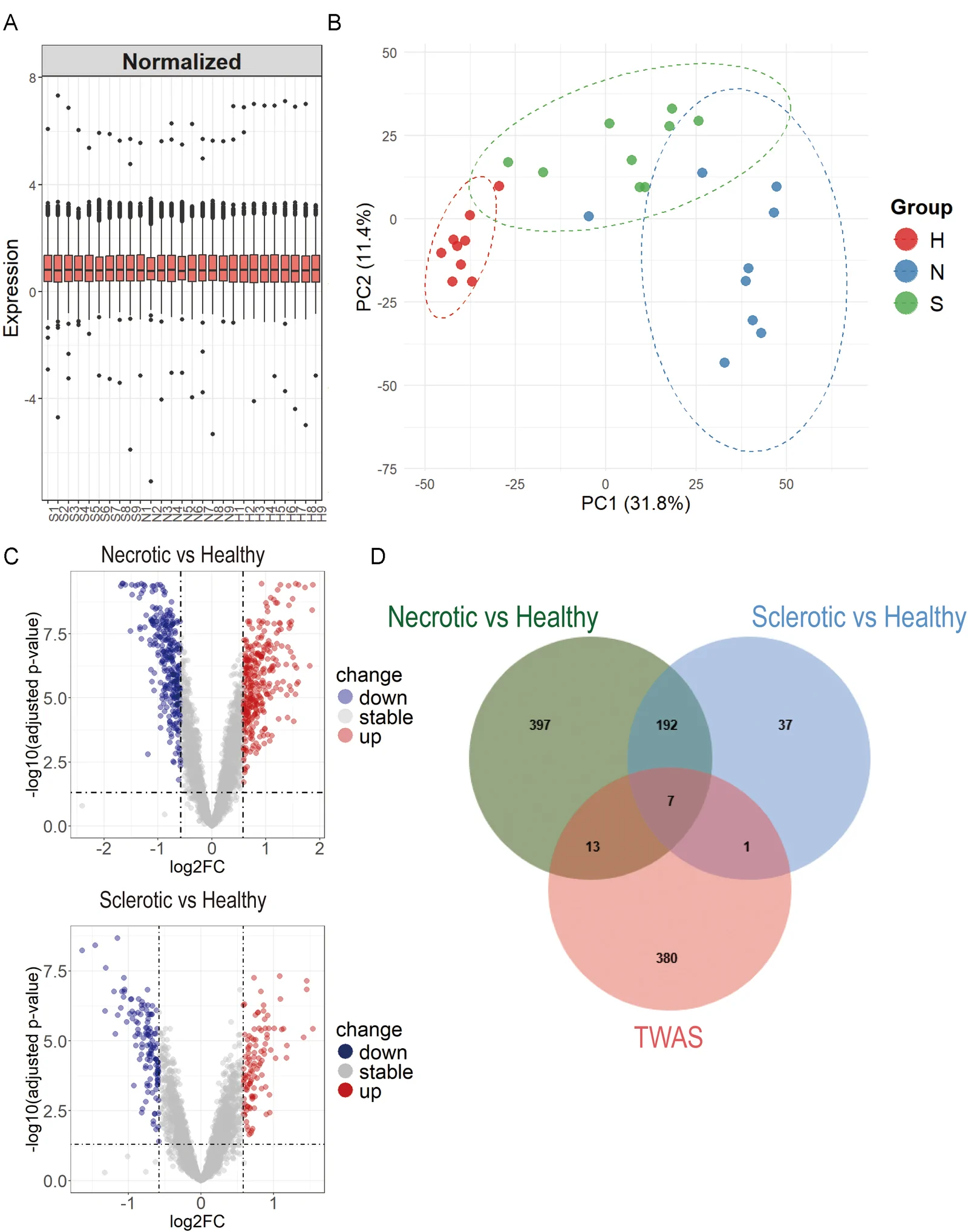
Analysis of differentially expressed proteins (DEPs) and prioritization of genetic candidates for MR analyses. (A) Boxplot of proteomic data following variance-stabilizing normalization. (B) PCA plot showing distinct clustering of necrotic, sclerotic, and healthy tissues. (C) Volcano plots illustrating DEPs: Top, necrotic vs. healthy (*n* = 609); bottom, sclerotic vs. healthy (*n* = 237). Red points indicate upregulated proteins; blue points indicate downregulated proteins. (D) Venn diagram showing the 21 genes overlapping between TWAS and proteomics results, prioritized for MR analysis.

Differential expression analysis, performed using the *limma* package, compared necrotic versus healthy and sclerotic versus healthy tissues under stringent thresholds set at |log2FC| ≥ 0.58 and adj.*p* < 0.05. In the necrotic–healthy comparison, 609 DEPs were detected (313 upregulated, 296 downregulated). In the sclerotic–healthy comparison, 237 DEPs were identified (111 proteins upregulated, 126 downregulated) (Supplementary Table S1 in Supplementary File 1) (Figure 3C).

### 3.3. Integration of TWAS and Proteomic Data

TWAS results retrieved from the TWAS Atlas identified 401 genes associated with osteonecrosis through FUSION-based analysis [20]. Cross-referencing these genes with our DEPs using Venn diagram analysis yielded 21 overlapping genes for subsequent MR analyses (Figure 3D).

Among these, seven genes (*GALK1*, *LIPA*, *STEAP4*, *ACADS*, *ALDH7A1*, *AKR1C2*, and *ACOT1*) were consistently dysregulated in both necrotic–healthy and sclerotic–healthy comparisons, suggesting involvement in both active necrosis and reparative processes. A single gene, *SYNM,* was uniquely dysregulated in the sclerotic–healthy comparison, implying roles in tissue remodeling. Thirteen genes (*S100A12*, *PPIC*, *GOLM1*, *GCA*, *HSPH1*, *CRLF3*, *TSC22D4*, *ATP5MF*, *DAG1*, *ALDH2*, *CAMK2D*, *ALDH1A1*, and *MDH1*) showed necrosis-specific expression changes, consistent with roles in early or progressive stages of bone tissue death.

### 3.4. Mendelian Randomization Results

By utilizing data from different sources and performing multiple analyses, we obtained a total of four sets of results for whole blood tissue and two sets for musculoskeletal tissue (Supplementary Tables S2–S7 in Supplementary File 1). After screening based on IVW significance was applied and associations due to pleiotropy or heterogeneity were excluded, 10 genes in whole blood and four genes in musculoskeletal tissue emerged with robust MR evidence supporting causal links to osteonecrosis (Supplementary Tables S8–S9 in Supplementary File 1).

To ensure the reliability of the MR results, we further filtered genes on the basis of consistency in the direction of effect across methods. All results demonstrated consistency across different analytical approaches (IVW, WME, and MR–Egger). Five genes in whole blood (*AKR1C2*, *ALDH2*, *TSC22D4*, *CAMK2D*, and *SYNM*) were excluded because of contradictory effect directions in IVW estimates across different analyses. After duplicates between whole blood and musculoskeletal tissues were removed, a total of seven genes were ultimately identified as candidate causal biomarkers for NONFH: *ACOT1*, *ALDH1A1*, *DAG1*, *STEAP4*, *LIPA*, *PPIC*, and *TSC22D4* (Table 2).

**Table 2.** Significant IVW results of MR analysis.

Gene Symbol	Significant IVW			Directional Consistency in Different Methods	Directional consistency in multiple analyses	Tissue	Effect
	No. of IVs	β	*p*				
*ACOT1*	38	–5.39 E–04	9.70 E–06	yes (3/3)	yes (4/4)	blood	protective
*AKR1C2*	101	–1.40 E–03	2.39 E–03	yes (3/3)	no (1/2)	blood	protective
*ALDH1A1*	106	–3.14 E-04	4.28 E–08	yes (3/3)	yes (4/4)	blood	protective
*ALDH2*	99	3.72 E–04	8.70 E–18	yes (3/3)	no (2/4)	blood	risk-increasing
*DAG1*	9	1.13 E–03	6.97 E–05	yes (3/3)	yes (2/2)	blood	risk-increasing
*TSC22D4*	35	8.47 E–04	3.08 E–08	yes (3/3)	no (1/2)	blood	risk-increasing
*CAMK2D*	55	–1.38 E–01	1.68 E–03	yes (3/3)	no (1/2)	blood	protective
*STEAP4*	134	1.43 E–01	6.45 E–10	yes (3/3)	yes (2/2)	blood	risk-increasing
*SYNM*	7	3.61 E–04	1.46 E–04	yes (3/3)	no (2/4)	blood	risk-increasing
*LIPA*	4	1.41 E–01	1.51 E–03	yes (3/3)	yes (4/4)	blood	risk-increasing
*ACOT1*	16	–2.50 E–04	1.04 E–06	yes (3/3)	yes (2/2)	bone	protective
*PPIC*	11	2.71 E–04	6.23 E–04	yes (3/3)	yes (2/2)	bone	risk-increasing
*TSC22D4*	7	3.60 E–04	1.14 E-04	yes (3/3)	yes (2/2)	bone	risk-increasing
*LIPA*	4	1.81 E–01	4.77 E–03	yes (3/3)	yes (2/2)	bone	risk-increasing

In whole blood, *ACOT1*, *ALDH1A1*, *DAG1*, *STEAP4*, and *LIPA* demonstrated significant causal associations. In musculoskeletal tissue, *ACOT1*, *PPIC*, *TSC22D4*, and *LIPA* were implicated. *ACOT1* and *ALDH1A1* exhibited protective effects (*β* < 0), whereas *DAG1*, *STEAP4*, *LIPA*, *PPIC*, and *TSC22D4* were risk-increasing (*β* > 0).

### 3.5. Validation of Biomarker Effects

We first confirmed that MR-derived causal effects were directionally consistent with corresponding TWAS effect estimates for all genes (Supplementary File 1, Table S10).

We then visualized protein abundance across bone tissue types via heatmaps (Figure 4). *ACOT1* and *ALDH1A1*, protective in MR analyses, were more abundant in healthy and sclerotic tissues than in necrotic regions. Conversely, *LIPA*, *PPIC*, and *TSC22D4*, risk-enhancing in MR analyses, showed higher abundance in necrotic tissue. Notably, *DAG1* and *STEAP4*, although classified as risk-enhancing genes in MR, displayed reduced protein abundance in necrotic tissue, suggesting possible context-dependent or temporally dynamic expression patterns.

**Figure 4.**
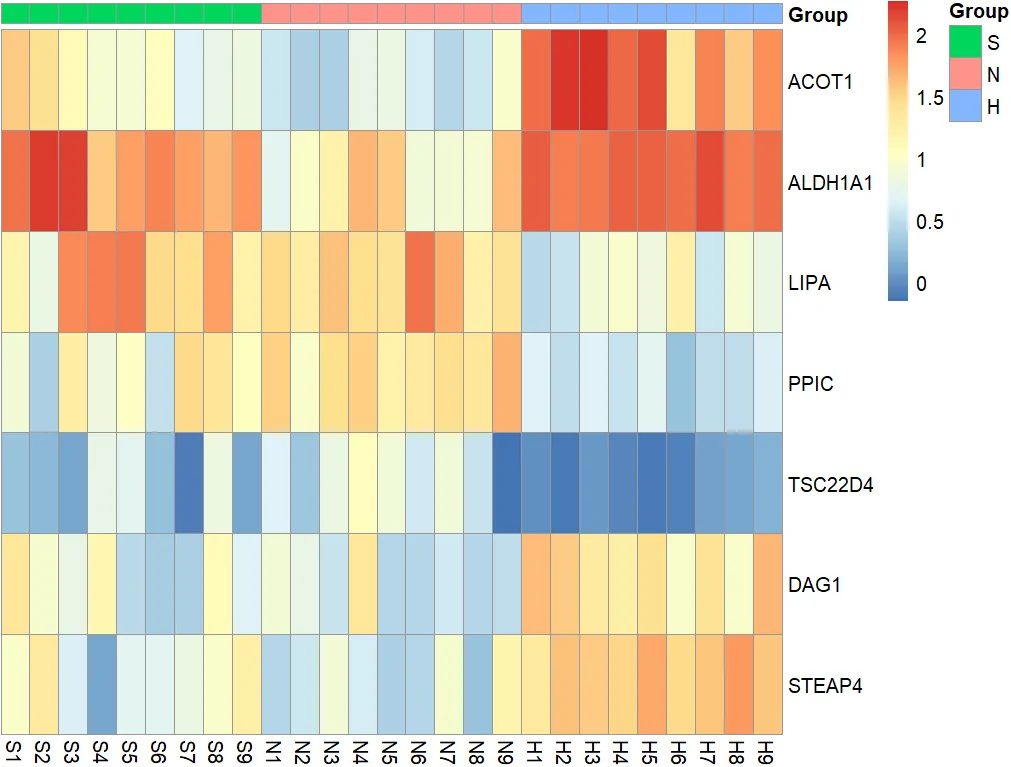
Regional protein expression patterns for MR-identified candidate biomarkers. Heatmap illustrating relative protein abundance (orange: upregulated; blue: downregulated) across healthy, sclerotic, and necrotic tissues. Protective biomarkers *ACOT1* and *ALDH1A1* are enriched in healthy and sclerotic tissues, whereas risk biomarkers *LIPA*, *PPIC*, and *TSC22D4* are enriched in necrotic tissue. *DAG1* and *STEAP4* show discordance between MR-predicted risk and observed protein distribution.

Notably, both *ACOT1* and *LIPA* showed significant results in MR analyses using both whole blood and musculoskeletal tissue data, and their effect directions were consistent with inter-regional protein expression patterns, supporting higher confidence in these two genes. Additionally, given that musculoskeletal tissue is more directly related to the disease, we subsequently performed single-cell sequencing analysis on the four genes identified in musculoskeletal tissue (i.e., *ACOT1*, *PPIC*, *TSC22D4*, and *LIPA*).

### 3.6. Single-Cell Transcriptomic Analysis

In total, 52,322 cells from OA (*n* = 17,611), ARCO stage III (*n* = 14,621), and ARCO stage IV (*n* = 20,090) groups were clustered into 24 groups, aggregated into 12 major cell types: T cells, fibroblasts, endothelial cells, monocytes, B cells, reticular cells, granulocytes, vascular smooth muscle cells (VSMCs), erythroid cells, osteoblasts, osteoclasts, and mast cells (Figure 5A). The rationale for cell annotation and expression levels of classical markers is shown in Supplementary File 2, Figure S1.

**Figure 5.**
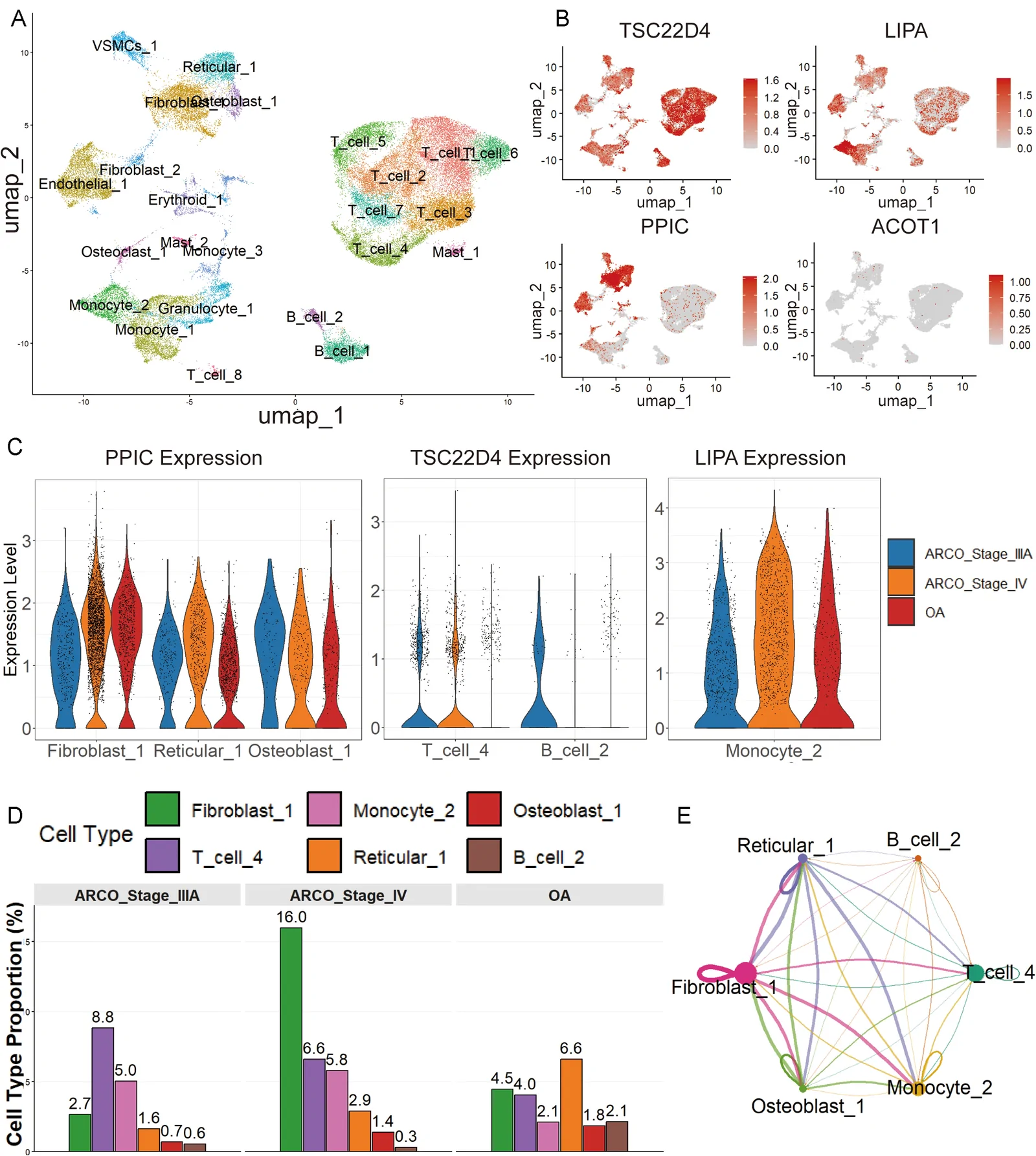
Single-cell transcriptomics of musculoskeletal biomarkers. (A) UMAP embedding of 52,322 cells, grouped into 24 clusters and annotated into 12 major lineages. (B) UMAP plots showing distribution of *TSC22D4*, *PPIC*, and *LIPA* expression. (C) Violin plots showing cell type-specific expression of *TSC22D4*, *PPIC,* and *LIPA* in OA, ARCO III, and ARCO IV. (D) Proportional representation of six key subpopulations across disease groups. (E) Circle plot of intercellular communication among the six subpopulations. Dot size represents cell count; edge thickness indicates interaction strength.

We examined the four musculoskeletal MR biomarkers at single-cell resolution. *ACOT1* showed minimal expression across clusters; *TSC22D4* was enriched in T_cell_4 and B_cell_2; *LIPA* was predominant in Monocyte_2; *PPIC* was highly expressed in Fibroblast_1, Reticular_1, and Osteoblast_1 (Figure 5B, Supplementary File 2 Figure S2). Disease comparisons revealed that (1) *PPIC* expression in Reticular_1 was increased in NONFH versus OA; (2) *TSC22D4* expression was highly specific to T_cell_4 and B_cell_2 in NONFH while being largely absent in OA; and (3) *LIPA*-high Monocyte_2 cells were more frequent in ARCO stage IV than in OA (Figure 5C). The alignment of these patterns with MR-predicted pathogenicity strengthens evidence for their functional involvement in NONFH.

Population proportions analysis revealed that Fibroblast_1 was notably expanded in ARCO stage IV (Figure 5D). Focused cell–cell communication analysis between ARCO III and IV highlighted highly active signaling between Fibroblast_1 and five other subpopulations (Figure 5E). Among the top enriched signaling pathways (Supplementary File 1 Table S11), COLLAGEN and MIF signaling predominantly originated from Fibroblast_1, whereas MK signaling originated mainly from Reticular_1 (Supplementary File 2 Figure S3).

## 4. Discussion

In this study, we employed an integrative analytical framework combining TWASs and MR with DIA-based proteomics to identify causal biomarkers and potential therapeutic targets for NONFH. This multi-omics approach offers several advantages over conventional proteomic analyses: it filters DEPs for experimental validation; prioritizes candidates with genetic evidence for causality; mitigates confounding and reverse causation through MR; facilitates the discovery of novel biomarkers through genetic–protein associations; and leverages tissue-specific eQTLs to enhance translational relevance, which is particularly relevant to a disease such as NONFH, where pathophysiology is likely to differ between systemic circulation and local bone tissue.

### 4.1. Key Causal Biomarkers

Our integrated analysis revealed *ACOT1* (acyl-CoA thioesterase 1) and *LIPA* (lysosomal acid lipase) as particularly noteworthy because their genetically predicted expression was causally associated with NONFH in both blood and musculoskeletal tissues. *ACOT1*, localized to the cytoplasm, catalyzes the hydrolysis of acyl-CoA to free fatty acids and CoA-SH, a critical process in lipid metabolism [21]. Additionally, *ACOT1* modulates *PPARα* signaling [22]. Previous transcriptomic data suggest upregulation of *ACOT1* in bone marrow cells of ketogenic diet-induced osteoporotic mice [23], implicating it in bone lipid metabolism and energy homeostasis. In our MR analysis, elevated *ACOT1* expression exerted a protective effect against NONFH, suggesting that its lipid-regulatory function may counteract metabolic disturbances implicated in osteonecrosis.

In contrast, *LIPA*, an enzyme responsible for the hydrolysis of cholesteryl esters and triglycerides in the lysosome [24, 25], was associated with increased risk. Its overexpression has been linked to immune cell infiltration and pro-inflammatory responses [26], whereas deficiency impairs osteoblast proliferation and bone repair [27]. These dual biological roles (i.e., promoting inflammation yet supporting bone formation) highlight the need for context-specific evaluation of *LIPA* in NONFH.

### 4.2. Tissue-Specific Risk Biomarkers

Two additional musculoskeletal tissue-specific biomarkers, *PPIC* (peptidyl-prolyl cis-trans isomerase C) and *TSC22D4* (TSC22 domain family protein 4), exhibited risk-increasing effects. Although the role of *PPIC* is not fully understood, its function in protein folding [28] could influence the stability of structural or signaling proteins essential for bone homeostasis. *TSC22D4* is a negative regulator of lipogenic gene expression [29]; its deletion alleviates hepatic lipid accumulation and inflammation [30]. Given that lipid metabolism dysregulation and fat embolism have been implicated in NONFH pathogenesis [31], the observed association with *TSC22D4* may reflect pathogenic contributions via metabolic and vascular pathways.

In blood, *ALDH1A1* (aldehyde dehydrogenase 1A1) emerged as a protective biomarker. Interestingly, in vitro studies indicate that *ALDH1A1* deficiency enhances osteogenic differentiation and adipogenesis of MSCs [32]. Although higher *ALDH1A1* expression could potentially impede bone repair in NONFH, its inhibition of marrow adiposity may offset risk by reducing intramedullary fat and circulatory impairment. This duality emphasizes the complexity of translating expression changes into functional outcomes in NONFH.

### 4.3. Discordant Cases and the Necrotic Environment

The complex necrotic bone microenvironment in NONFH, which is characterized by osteocyte apoptosis, marrow edema, ischemic conditions, and inflammatory cell infiltration [33], can confound the relationship between protein abundance and causal effects inferred from MR. This may explain the apparent discordance observed for *DAG1* (dystroglycan 1) and *STEAP4* (metalloreductase STEAP4), which were classified as risk-increasing in MR but showed reduced expression in necrotic tissue. In particular, *STEAP4* regulates iron uptake and utilization in osteoclasts, where its downregulation can suppress osteoclastogenesis [34]. Such discrepancies could arise from temporal expression shifts, post-translational regulation, or tissue remodeling dynamics.

### 4.4. Single-Cell Context and Cellular Niches

Our single-cell RNA sequencing analysis further contextualized four causal musculoskeletal biomarkers (*ACOT1*, *TSC22D4*, *PPIC*, and *LIPA*) within specific cell subpopulations, revealing disease-relevant expression in fibroblasts, osteoblasts, reticular cells, monocytes, and distinct B/T cell subsets. Notably, *PPIC* was enriched in Fibroblast_1, Reticular_1, and Osteoblast_1; *TSC22D4* was restricted to T_cell_4 and B_cell_2 in NONFH compared to OA; and *LIPA* was abundant in Monocyte_2, with disease-specific enrichment patterns. These cell populations have previously been implicated in NONFH pathogenesis through T-cell dysregulation [35] and osteoblasts apoptosis [36], underscoring their potential as pathogenic niches.

Cell–cell communication analysis identified the COLLAGEN and MIF pathways (predominantly originating from Fibroblast_1) and MK signaling (from Reticular_1) as possible mediators of interpopulation crosstalk in advanced disease stages. These findings provide a new cellular framework for assessing how causal biomarkers operate within multicellular networks, potentially influencing both tissue destruction and repair.

### 4.5. Methodological Strengths and Considerations

By integrating TWAS, MR, tissue-specific eQTLs, proteomics, and scRNA-seq, this study provides a multi-layered prioritization strategy for candidate biomarkers. The use of independent replication across different tissue data sources and stringent statistical thresholds strengthens confidence in the identified associations. However, functional validation in experimental models remains a critical next step, especially to clarify biomarkers with discordant genetic and proteomic signals. Additionally, given the modest sample size for proteomics, replication in larger, independent cohorts is warranted to ensure generalizability.

## 5. Conclusion

Using a novel integrative TWAS–MR–proteomics pipeline, we identified seven causal biomarkers for NONFH: *ACOT1*, *LIPA*, *PPIC*, *TSC22D4*, *ALDH1A1*, *DAG1*, and *STEAP4*. These genes represent largely underexplored candidates in the context of NONFH pathogenesis. These biomarkers span both systemic and local musculoskeletal contexts, reflecting the multifaceted pathophysiology of NONFH, and may influence disease progression through diverse mechanisms including lipid metabolism, inflammation, extracellular matrix remodeling, and intercellular signaling.

Our findings suggest that *ACOT1* and *ALDH1A1* may confer protective effects, whereas *LIPA*, *PPIC*, *TSC22D4*, *DAG1*, and *STEAP4* are risk-enhancing, with the latter group offering promising targets for therapeutic intervention. The incorporation of single-cell transcriptomic mapping provides an additional layer of insight into the specific cellular niches where these biomarkers may operate.

Future work should focus on functional characterization of these biomarkers in relevant cellular and animal models, longitudinal patient studies to track biomarker dynamics over disease progression, and clinical validation to assess their diagnostic and prognostic potential. The tissue-specific nature observed for some biomarkers underscores the importance of considering both local bone tissue and systemic factors in developing targeted strategies for early detection and treatment of NONFH.

In conclusion, this study provides a robust foundation for a more mechanistic and precision-oriented approach to NONFH research, highlighting promising biomarkers that may significantly advance our understanding of disease biology and opening avenues for biomarker-driven diagnosis and therapy.

## Supplementary Material

Supplementary File 1

Supplementary File 2

## Data Availability

Data from Study Deposited in a Repository and Available The data generated and analyzed during this study are available in the ProteomeXchange Consortium via the iProX partner repository (dataset identifier: PXD048708) at the following link: https://proteomecentral.proteomexchange.org. Used Public Data Data from the UK Biobank were derived from its GWAS data (Round 2) publicly released in August 2018 (https://www.nealelab.is/uk-biobank). Data from the FinnGen consortium were obtained from its published GWAS data (DF12) released in December 2024 (https://www.finngen.fi/en). This study utilized eQTL data from the eQTLGen consortium (https://eqtlgen.org/cis-eqtls.html), along with TWAS summary statistics from the TWAS Atlas database (https://ngdc.cncb.ac.cn/twas/browse#traits). Publicly available data from the GTEx Project (https://www.gtexportal.org/home/) were also employed in this research. The GTEx Project was supported by the Common Fund of the Office of the Director of the National Institutes of Health, as well as by NCI, NHGRI, NHLBI, NIDA, NIMH, and NINDS. These data were accessed using the xQTLbiolinks R package (https://dingruofan.github.io/xQTLbiolinks/index.html). The single-cell RNA sequencing data were obtained from publicly available data associated with the publication (DOI: 10.1038/s42003-022-03271-6)[19].

## Abbreviations

*ACOT1*: acyl‑coenzyme A thioesterase 1
*ALDH1A1*: aldehyde dehydrogenase 1A1
*DAG1*: dystroglycan 1
DDA: data-dependent acquisition
DEPs: differentially expressed proteins
DIA: data-independent acquisition
eQTLs: expression quantitative trait loci
FC: fold change
GWAS: genome-wide association study
iRT: indexed retention time
IVs: instrumental variables
IVW: inverse-variance weighted
*LIPA*: lysosomal acid lipase/cholesteryl ester hydrolase
MR: Mendelian randomization analyses
MR–Egger: Mendelian randomization–Egger regression
MS: mass spectrometry
NONFH: non-traumatic osteonecrosis of the femoral head
ONFH: osteonecrosis of the femoral head
*PPIC*: peptidyl-prolyl cis-trans isomerase C
scRNA-seq: single-cell RNA sequencing
SNPs: single nucleotide polymorphisms
*STEAP4*: metalloreductase STEAP4
THA: total hip arthroplasty
*TSC22D4*: TSC22 domain family protein 4
TWAS: transcriptome-wide association study
UMI: unique molecular identifiers
WME: weighted median estimator
