# Supplementary material for: Integrative Mendelian Randomization and Proteomic Analyses Reveal Genetically Supported Candidate Causal Biomarkers for Non-Traumatic Osteonecrosis of the Femoral Head
Source: J Mass Spectrom Adv Clin Lab. 2026 Jul 6;39(1):zrn0dg78. doi: 10.21627/zrn0dg78 (PMC13750208; doi:10.21627/zrn0dg78)
Supplement: Supplementary file 1 [file jmsacl-39-1-zrn0dg78-s002.docx]

**Supplementary Material**

Integrative Mendelian Randomization and Proteomic Analyses Reveal Genetically Supported Candidate Causal Biomarkers for Non-Traumatic Osteonecrosis of the Femoral Head

^‡^ Zhangzheng Wang, ^‡^Liang Mo, Wei He, Youwen Liu, Csaba Matta, Yuhao Liu

*** Corresponding Author(s):**
Yuhao Liu (doctor_yuhaoliu@126.com)

12 Jichang Road, Guangzhou, China

Csaba Matta (matta.csaba@med.unideb.hu)

Egyetem tér 1, H‑4032 Debrecen, Hungary

**Contents**

**Figure S1.** Cell Subpopulation Annotation and Classical Marker Expression.

**Figure S2.** DotPlot of Four Causal Biomarkers from Musculoskeletal Tissues Across Cell Subpopulations.

**Figure S3.** Chord diagram showing the sender–receiver relationships of major signaling pathways among six cell subpopulations.

**Figure S1.** Cell Subpopulation Annotation and Classical Marker Expression.

24 clusters were defined as 12 major cell subgroups: T cells (*CD3D*, *CD3E*, *CD4*), Fibroblasts (*COL1A1*, *DCN*), Endothelial cells (VWF, PECAM1, CLDN5), Monocytes (CD14, LYZ), B cells (*MS4A1*, *CD79A*), Reticular cells (*CXCL12*, *LEPR*), Granulocytes (*S100A8*), Vascular Smooth Muscle Cells (VSMCs) (*ACTA2*, *MYH11*, *TAGLN*), Erythroid cells (*GYPA*, *HBB*), Osteoblasts (*RUNX2*, *SP7*, *BGLAP*), Osteoclasts (*CTSK*, *ACP5*, *NFATC1*), and Mast cells (*KIT*, *TPSAB1*, *CPA3*).

**Figure S2.** DotPlot of Four Causal Biomarkers from Musculoskeletal Tissues Across Cell Subpopulations.

*TSC22D4* was predominantly highly expressed in T_cell_4 and B_cell_2, *LIPA* was mainly highly expressed in Monocyte_2, and *PPIC* was highly expressed in Fibroblast_1, Reticular_1, and Osteoblast_1. These six subpopulations were primarily included in the subsequent cell–cell communication analysis.

**Figure S3.** Chord diagram showing the sender–receiver relationships of major signaling pathways among six cell subpopulations.

Fibroblast_1 subpopulation acted as the primary sender for COLLAGEN and MIF signals, while Reticular_1 was the primary sender for MK signals.
